# Supplementary material for: Deep genome sequencing and variation analysis of 13 inbred mouse strains defines candidate phenotypic alleles, private variation and homozygous truncating mutations
Source: Genome Biol. 2016 Aug 1;17:167. doi: 10.1186/s13059-016-1024-y (PMC4968449; doi:10.1186/s13059-016-1024-y)
Supplement: Additional file 1: — Supplemental tables. Supplemental tables S1–S5. (PDF 109 kb) [file 13059_2016_1024_MOESM1_ESM.pdf]

Table s1. European Nucleotide Archive ERS IDs for all raw sequencing data for each strain. Jackson Laboratory stock numbers for each strain are also shown.

Strain	ERS ID	Stock number
**BUB/BnJ**	ERS661322	000653
**C57BL/10J**	ERS661323	000665
**C57BR/cdJ**	ERS661325	000667
**C58/J**	ERS661326	000669
**DBA/1J**	ERS661327	000670
**I/LnJ**	ERS661328	000674
**KK/HiJ**	ERS661329	002106
**MOLF/EiJ**	ERS661330	000550
**NZB/B1NJ**	ERS661331	000684
**NZW/LacJ**	ERS661332	001058
**RF/J**	ERS661333	000682
**SEA/GnJ**	ERS661334	000644
**ST/bJ**	ERS661335	000688

Table s2. Sensitivity estimates for all PCR validated structural variants.

Strain	Total PCR deletions	Missed	Sensitivity	Total PCR insertions	Missed	Sensitivity
**AKR/J**	132	9	93.18%	72	13	81.94%
**A/J**	149	11	92.62%	86	12	86.05%
**BALB/cJ**	138	9	93.48%	77	9	88.31%
**C3H/HeJ**	165	12	92.73%	93	11	88.17%
**CBA/J**	148	10	93.24%	87	5	94.25%
**DBA/2J**	151	12	92.05%	84	8	90.48%
**DBA/1J***	151	20	86.75%	84	16	80.95%
**LP/J**	136	8	94.12%	68	8	88.24%

*Sensitivity estimated for DBA/1J using the PCR validated structural variants identified in DBA/2J. When true structural variant differences between the strains are removed from the sensitivity analysis, the sensitivity increases to 91.6% (131/143) which is similar to the number of SNP sites that are identical (90.72%).

Table s3. Library and sequencing statistics for each sample.

Strain	Gender	Raw total sequences:	Average quality	Insert size average	Insert size standard deviation
**BUB/BnJ**	M	1344667465	35.8	256.3	92.7
**C57BL/10J**	M	1098531348	35	344.6	854.8
**C57BR/cdJ**	M	1404238082	34.1	258.1	109
**C58/J**	M	1526927921	36	262.7	123.9
**DBA/1J**	M	1394978119	35.1	254.6	110
**I/LnJ**	M	1243880652	35.7	217.4	82.4
**KK/HiJ**	M	1505723009	36.3	258.5	104.5
**MOLF/EiJ**	M	1119348103	35.7	194.7	68.5
**NZB/B1NJ**	M	1324550888	35.1	287.6	104.1
**NZW/LacJ**	M	1592592167	34.2	281.8	87.5
**RF/J**	M	1466072740	36.4	244.6	94.9
**SEA/GnJ**	M	1332757895	35.7	258.2	91.9
**ST/bJ**	M	2215849093	35.3	238.2	90.3

Table s4. Filter and cut-off values used to soft-filter SNPs and indels.

Filter	Description	Tag(s)	Value
**StrandBias**	Min P-value for strand bias	PV4	0.0001
**EndDistBias**	Min P-value for end distance bias	PV4	0.0001
**MaxDP**	Maximum read depth	DP or DP4	150-350*
**BaseQualBias**	Min P-value for baseQ bias	PV4	0
**MinMQ**	Minimum RMS mapping quality for SNPs	MQ	20
**MinAB**	Minimum number of alternate bases	DP4	5
**Qual**	Minimum value of the QUAL field	QUAL	10
**VDB**	Minimum Variant Distance Bias	VDB	0
**GapWin**	Window size for filtering adjacent gaps	-	3
**MapQualBias**	Min P-value for mapQ bias	PV4	0
**SnpGap**	SNP within INT bp around a gap to be filtered	-	2
**RefN**	Reference base is N	-	-
**MinDP**	Minimum read depth	DP or DP4	5
**Het**	Genotype call is heterozygous	low quality	-

*MaxDP calculated for each strain separately as 5 times the coverage and then rounded to the nearest 50.

Table s5. Parameters for each tool used to identify structural variation deletions and insertions.

Software	SV type	Version	Parameters*
**BreakDancerMax**	Deletion	1.1	bam2cfg.pl: -c 3 -n50000 -q 25<br>BreakDancer: -c3 -m 10000000 -q 25 -r 2 -y 25
**cnD**	Copy number losses and gains	1.3	--repeat-cut-off=0.35 --smooth=100
**LUMPY**	Deletion	0.2.11	extractSplitReads_BwaMem: default<br>extractDiscordants: samtools view -bh -F 1294<br>Lumpyexpress: -m 4 -r 0
**Scalpel**	Short novel sequence insertion	0.3.2	--window 600 --maxregcov 300
**Manta**	Novel sequence insertion	0.29.6	runWorkflow.py -m local
**Retroseq**	Transposable element insertion	1.5	Default

*A minimum of 10 support reads (read pairs or soft clipped reads) was required for all SVs to pass post-filtering
