# Supplementary material for: Deep genome sequencing and variation analysis of 13 inbred mouse strains defines candidate phenotypic alleles, private variation and homozygous truncating mutations
Source: Genome Biol. 2016 Aug 1;17:167. doi: 10.1186/s13059-016-1024-y (PMC4968449; doi:10.1186/s13059-016-1024-y)
Supplement: Additional file 2: — SNP, indel and SV density plots for all strains. SNP, indel and SV (deletions and insertions) density plots for all chromosomes (1–19, X and Y) for each strain. (PDF 3421 kb) [file 13059_2016_1024_MOESM2_ESM.pdf]

Chromosome 1

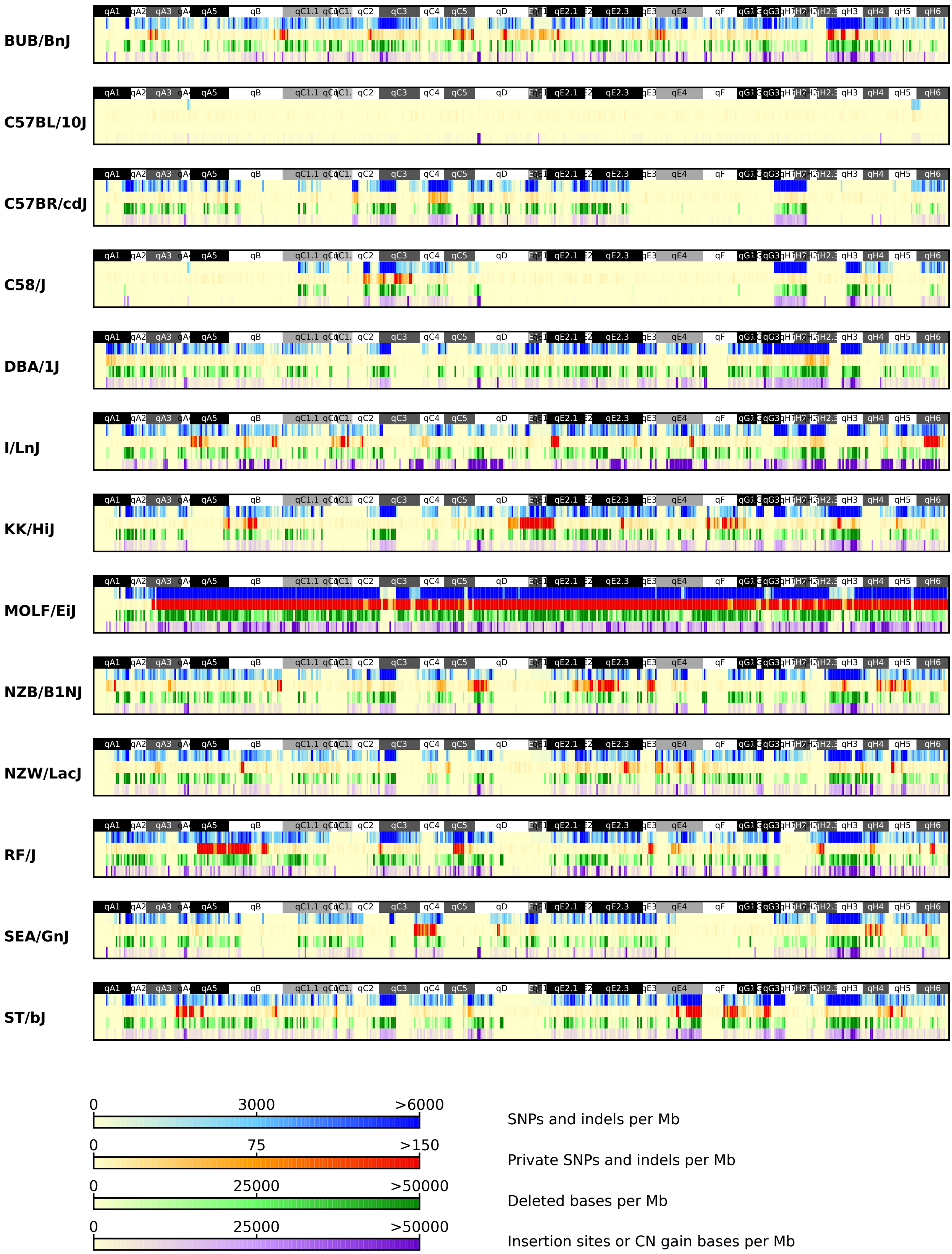

Chromosome 2

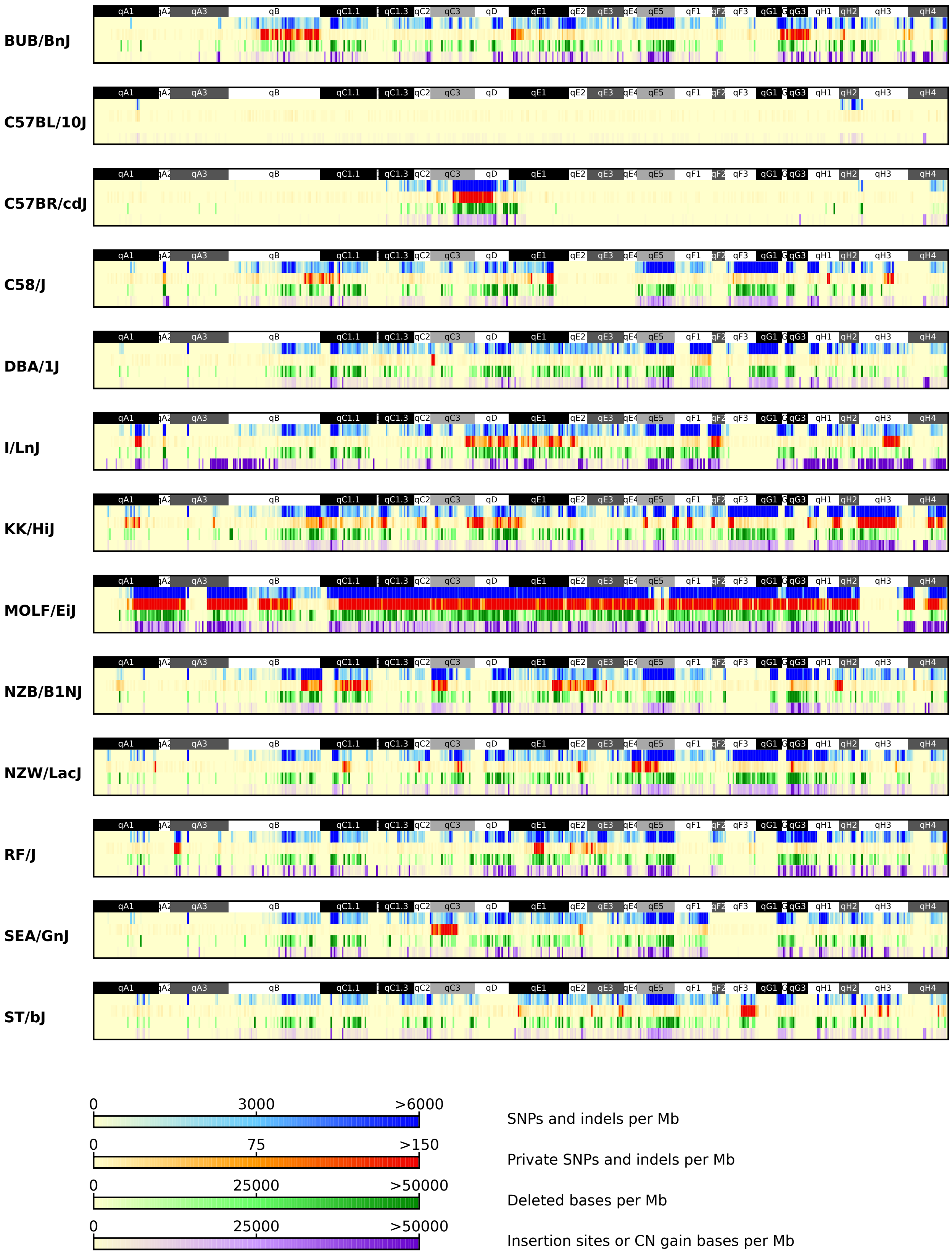

Chromosome 3

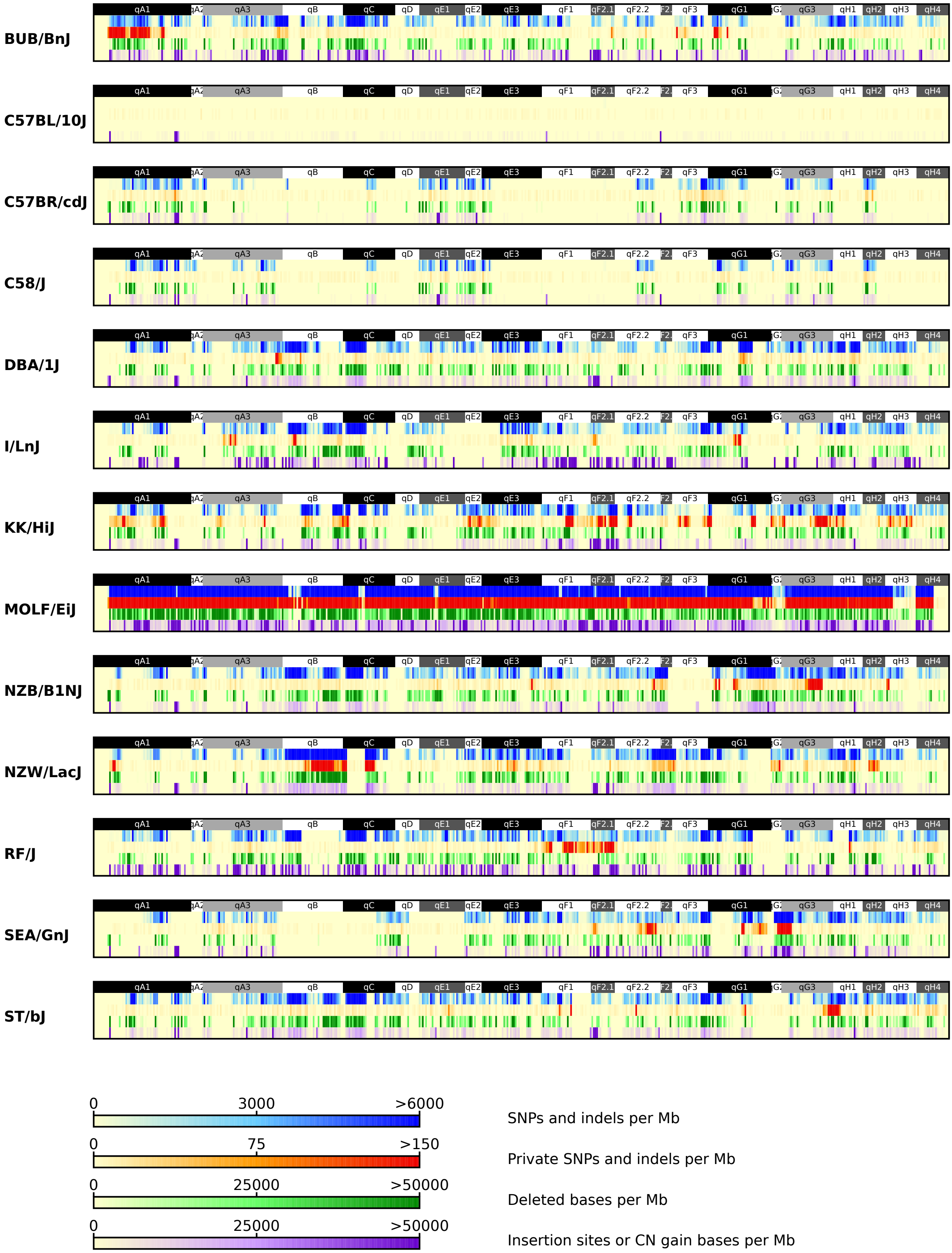

Chromosome 4

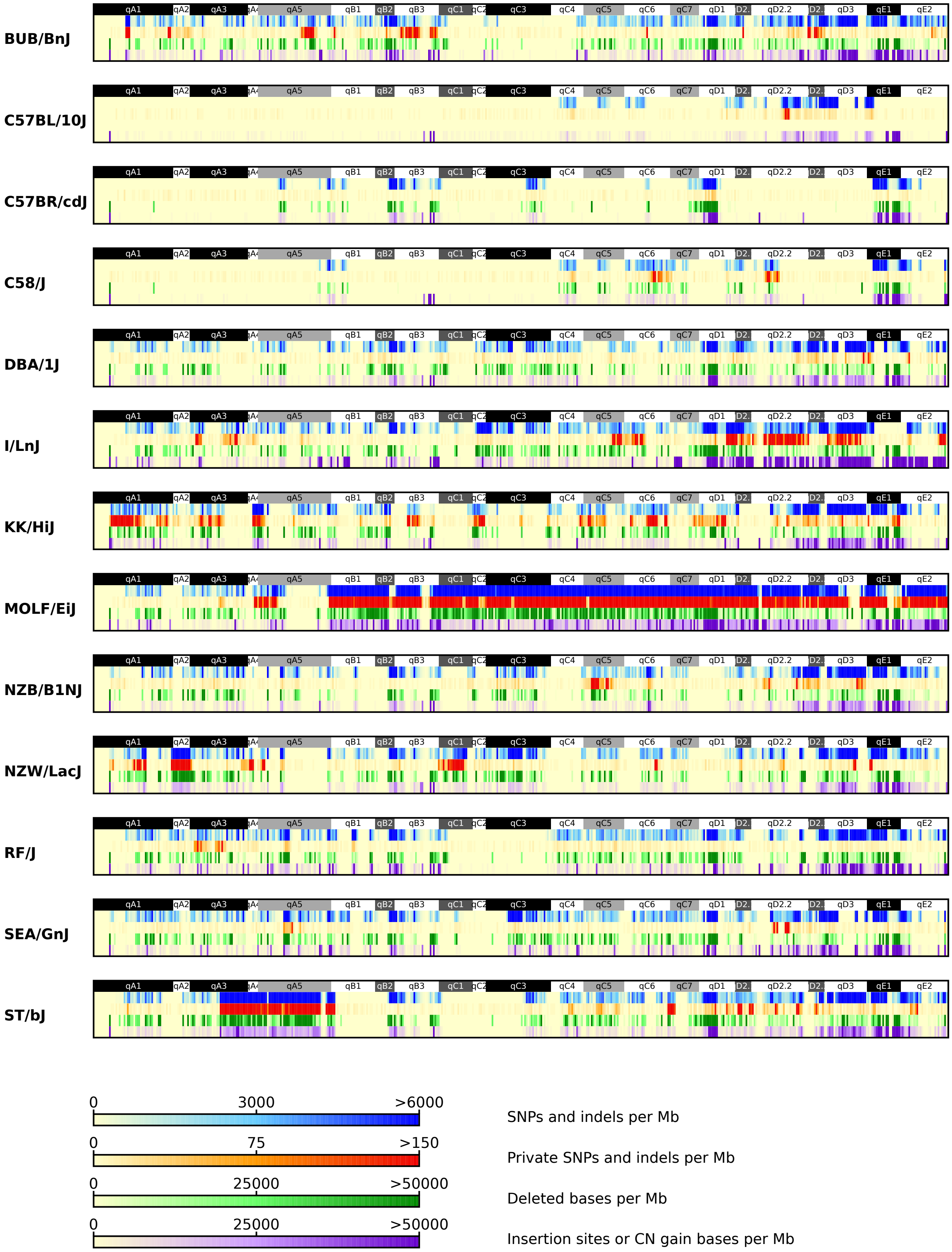

Chromosome 5

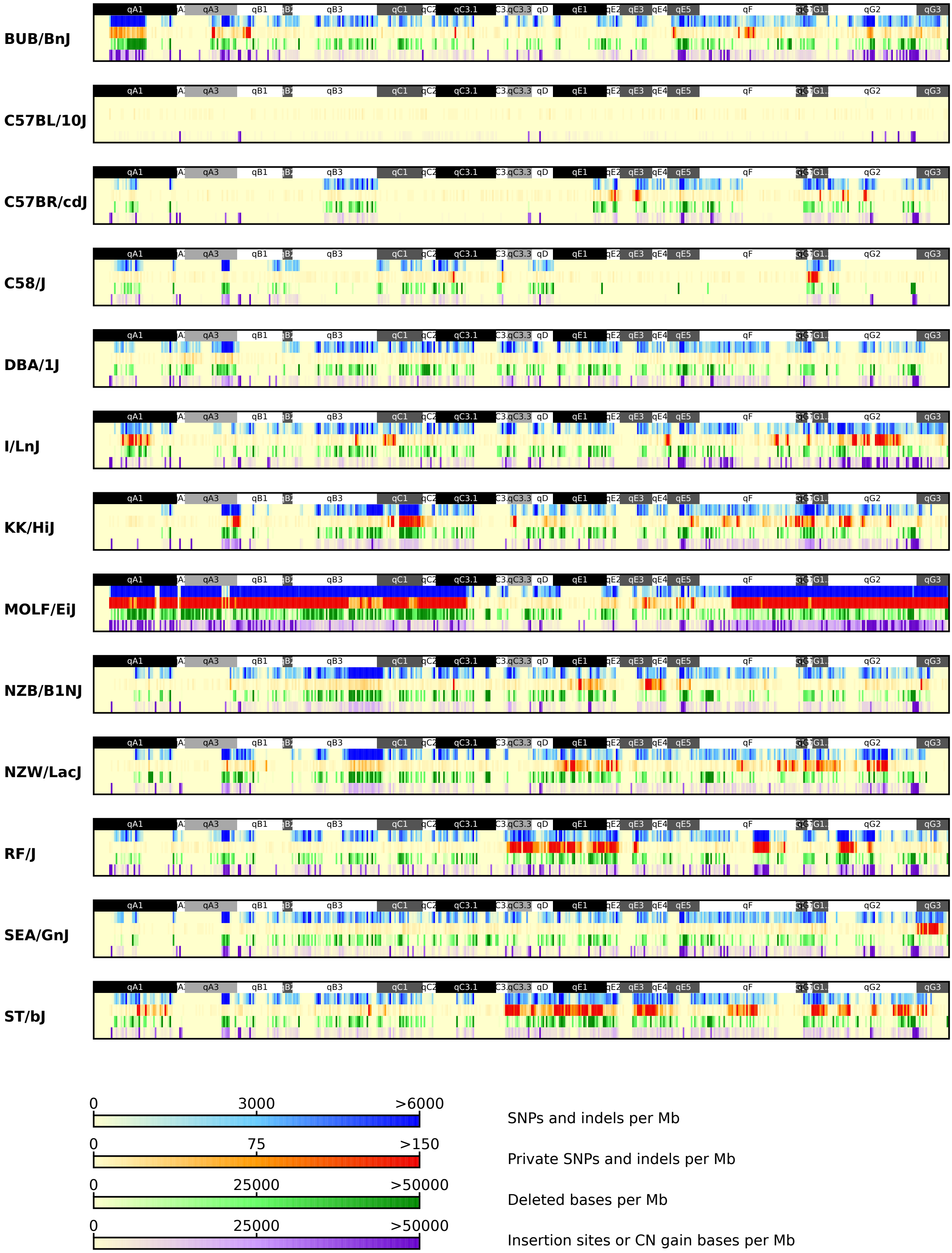

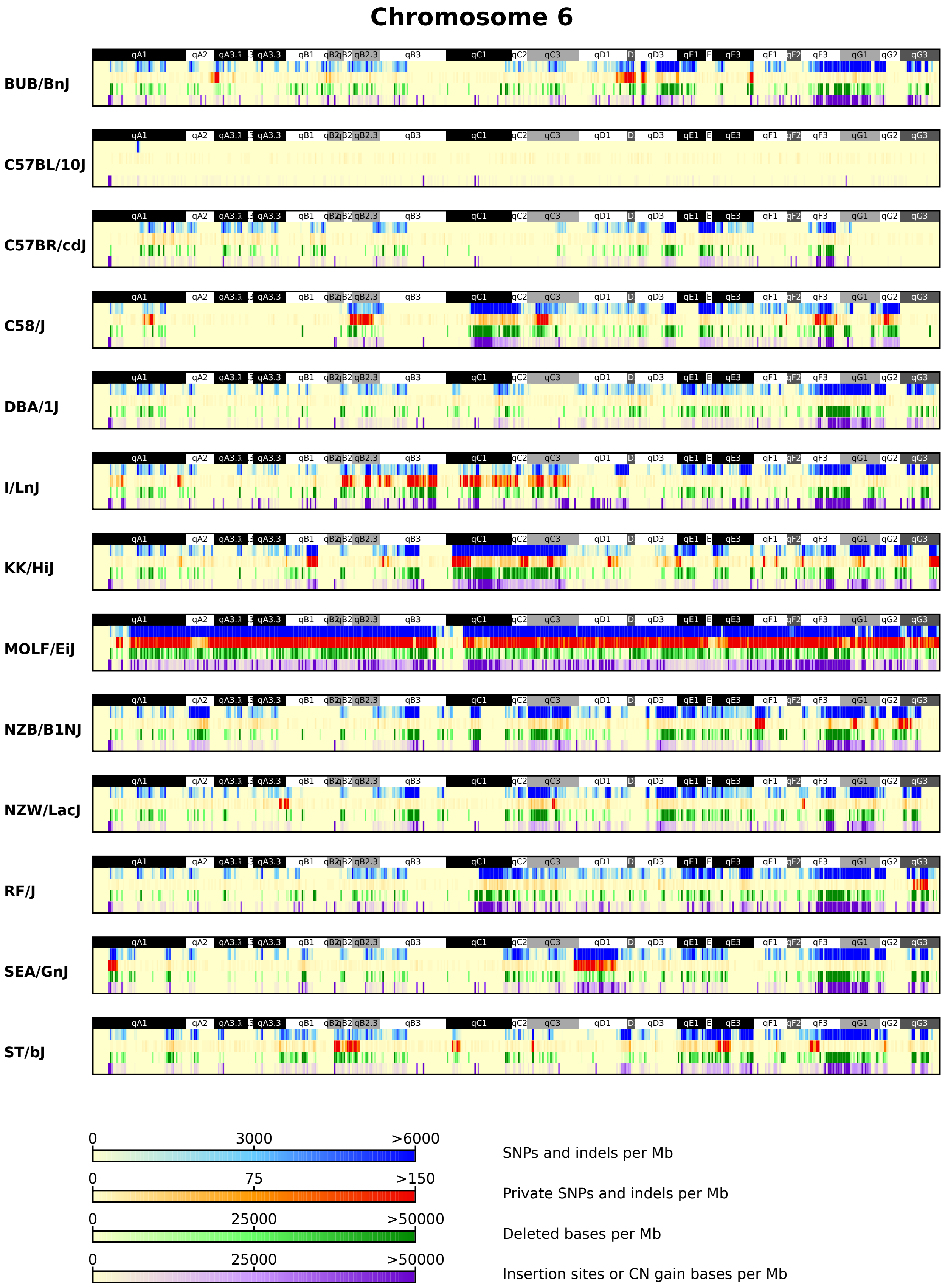
Chromosome 6
qA1 qA2 qA3.1 qA3.3 qB1 qB2.1 qB2 qB2.3 qB3 qC1 qC2 qC3 qD1 D qD3 qE1 E qE3 qF1 qF2 qF3 qG1 qG2 qG3
BUB/BnJ
C57BL/10J
C57BR/cdJ
C58/J
DBA/1J
I/LnJ
KK/HiJ
MOLF/EiJ
NZB/B1NJ
NZW/LacJ
RF/J
SEA/GnJ
ST/bJ
0 3000 >6000
SNPs and indels per Mb
0 75 >150
Private SNPs and indels per Mb
0 25000 >50000
Deleted bases per Mb
0 25000 >50000
Insertion sites or CN gain bases per Mb

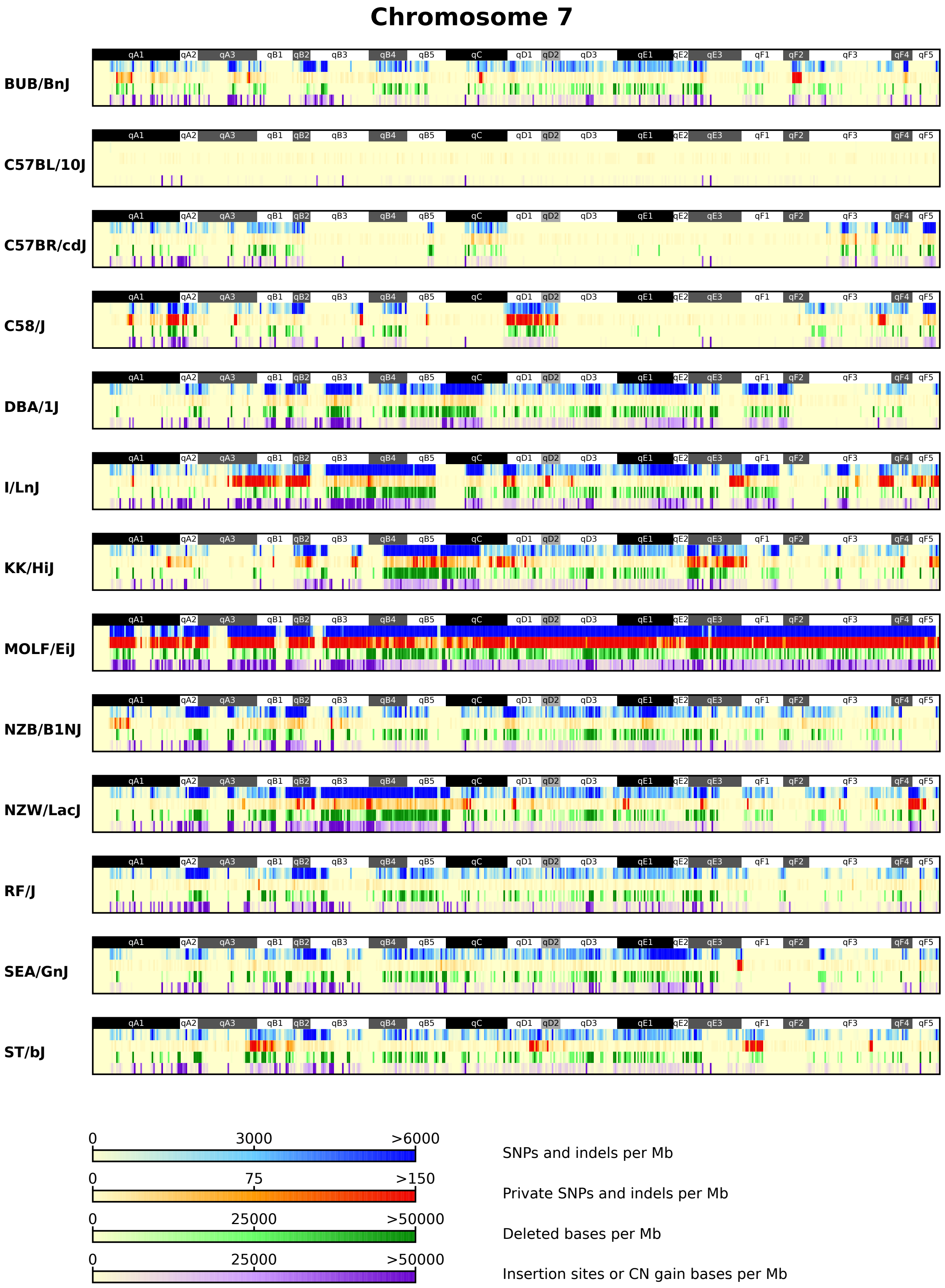

Chromosome 7
qA1
qA2
qA3
qB1
qB2
qB3
qB4
qB5
qC
qD1
qD2
qD3
qE1
qE2
qE3
qF1
qF2
qF3
qF4
qF5
BUB/BnJ
C57BL/10J
C57BR/cdJ
C58/J
DBA/1J
I/LnJ
KK/HiJ
MOLF/EiJ
NZB/B1NJ
NZW/LacJ
RF/J
SEA/GnJ
ST/bJ
0
3000
>6000
SNPs and indels per Mb
0
75
>150
Private SNPs and indels per Mb
0
25000
>50000
Deleted bases per Mb
0
25000
>50000
Insertion sites or CN gain bases per Mb

Chromosome 8

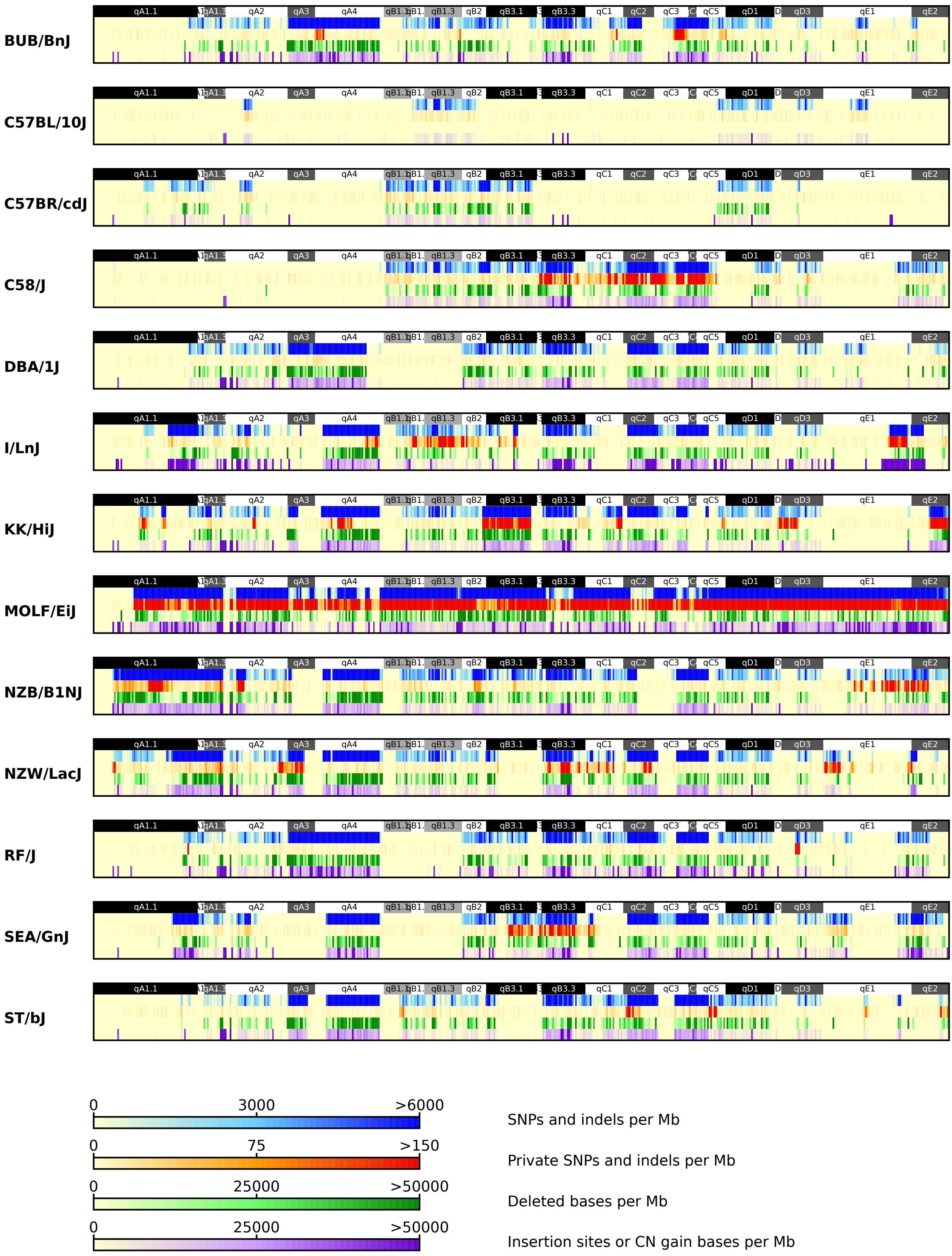

Chromosome 9

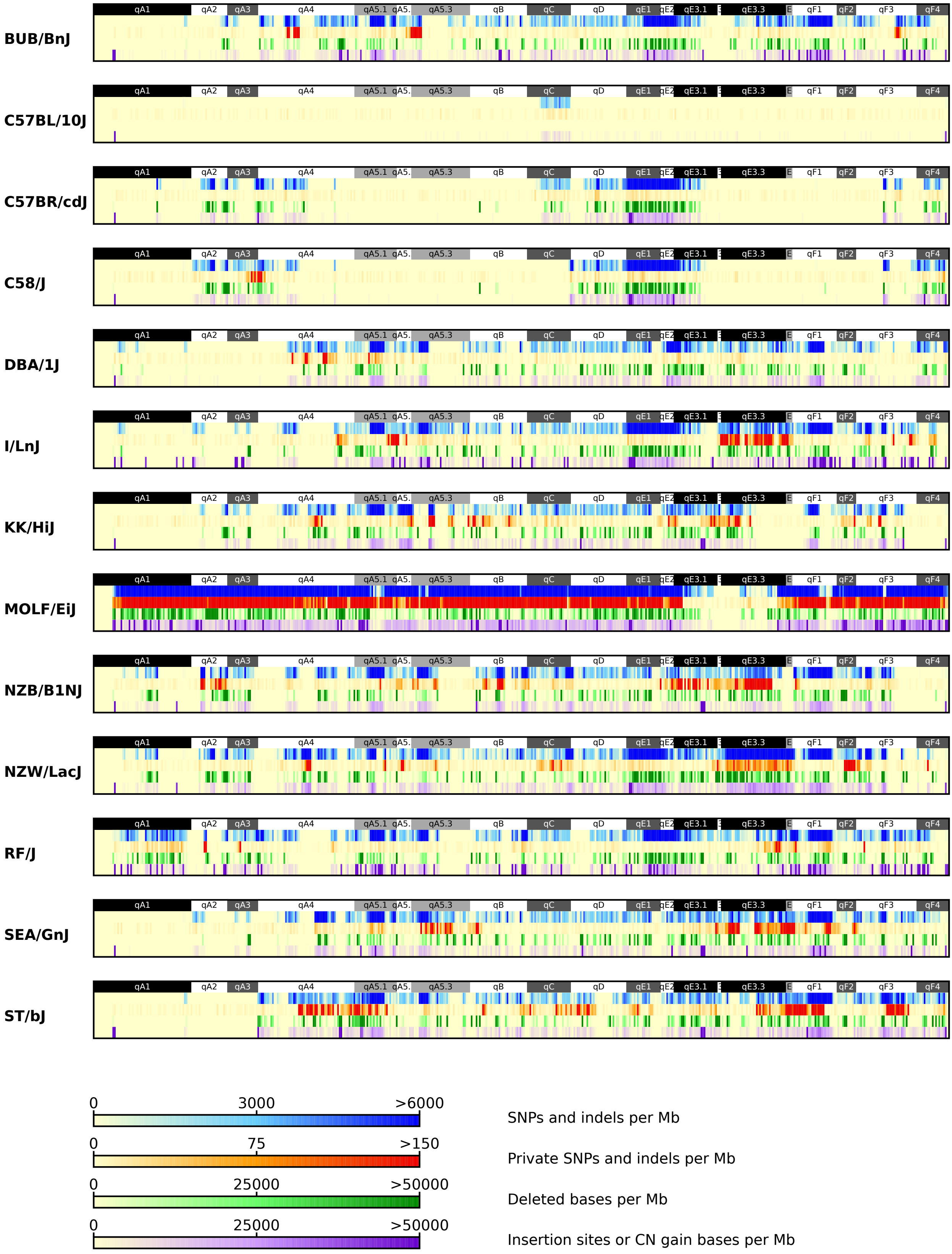

Chromosome 10

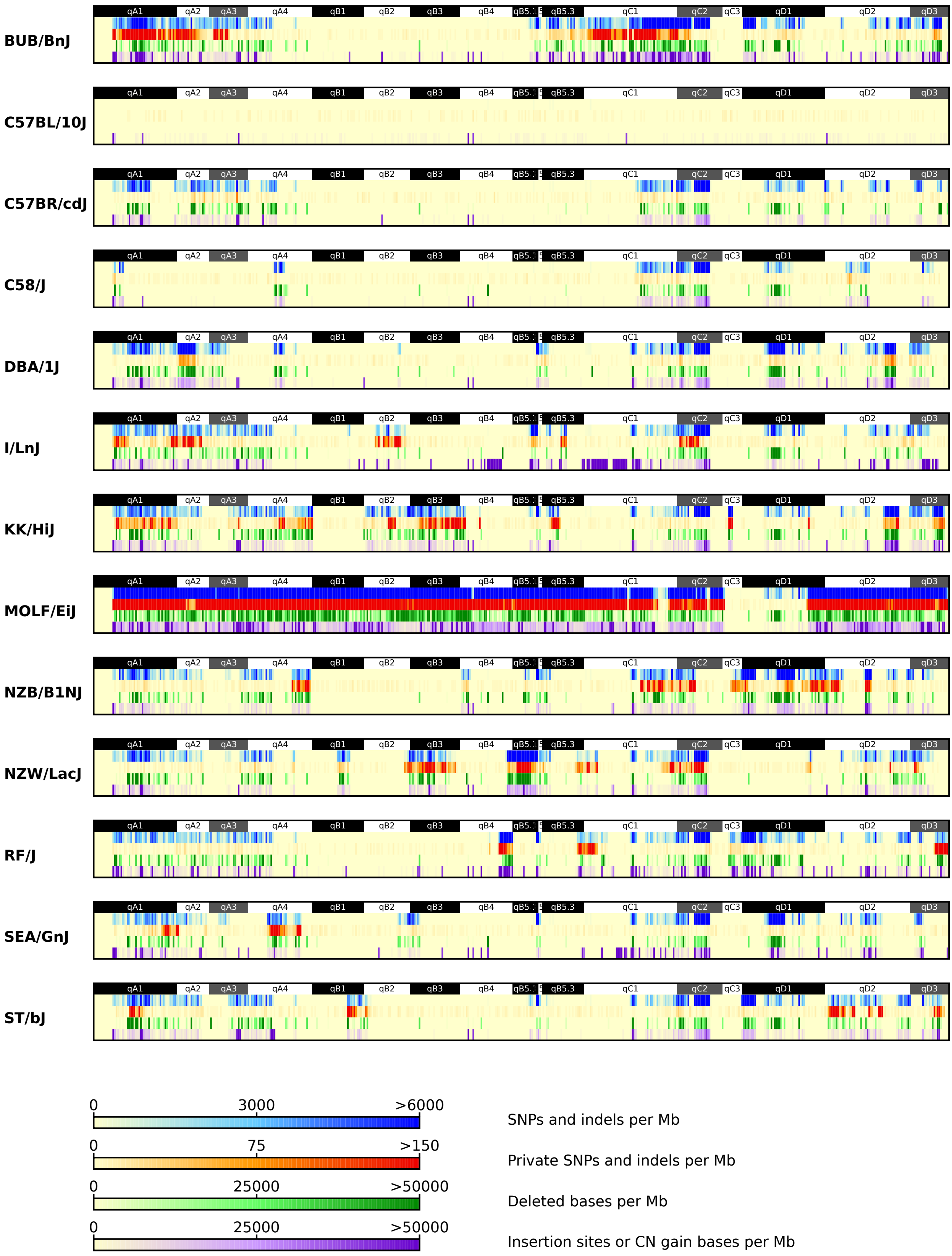

Chromosome 11

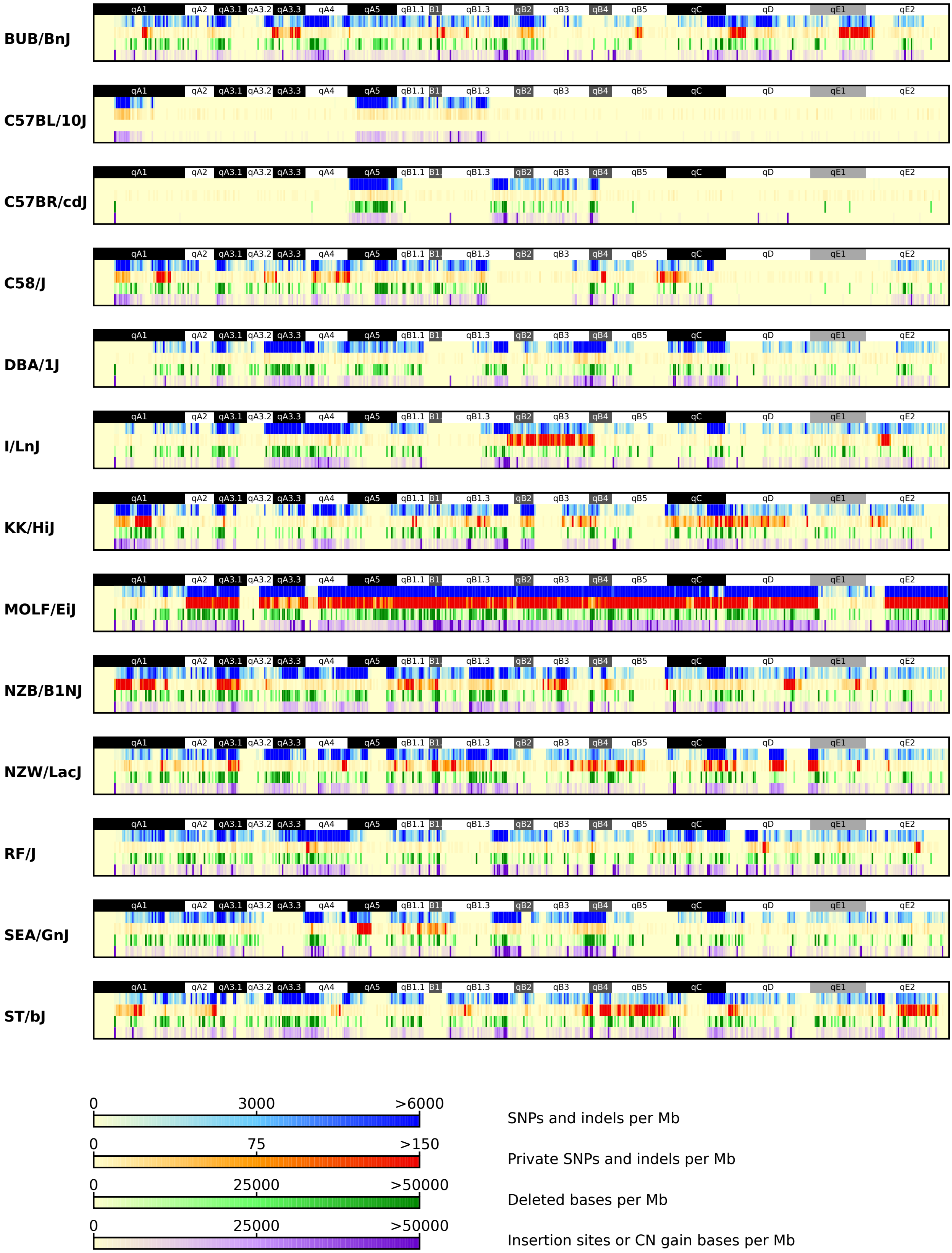

Chromosome 12

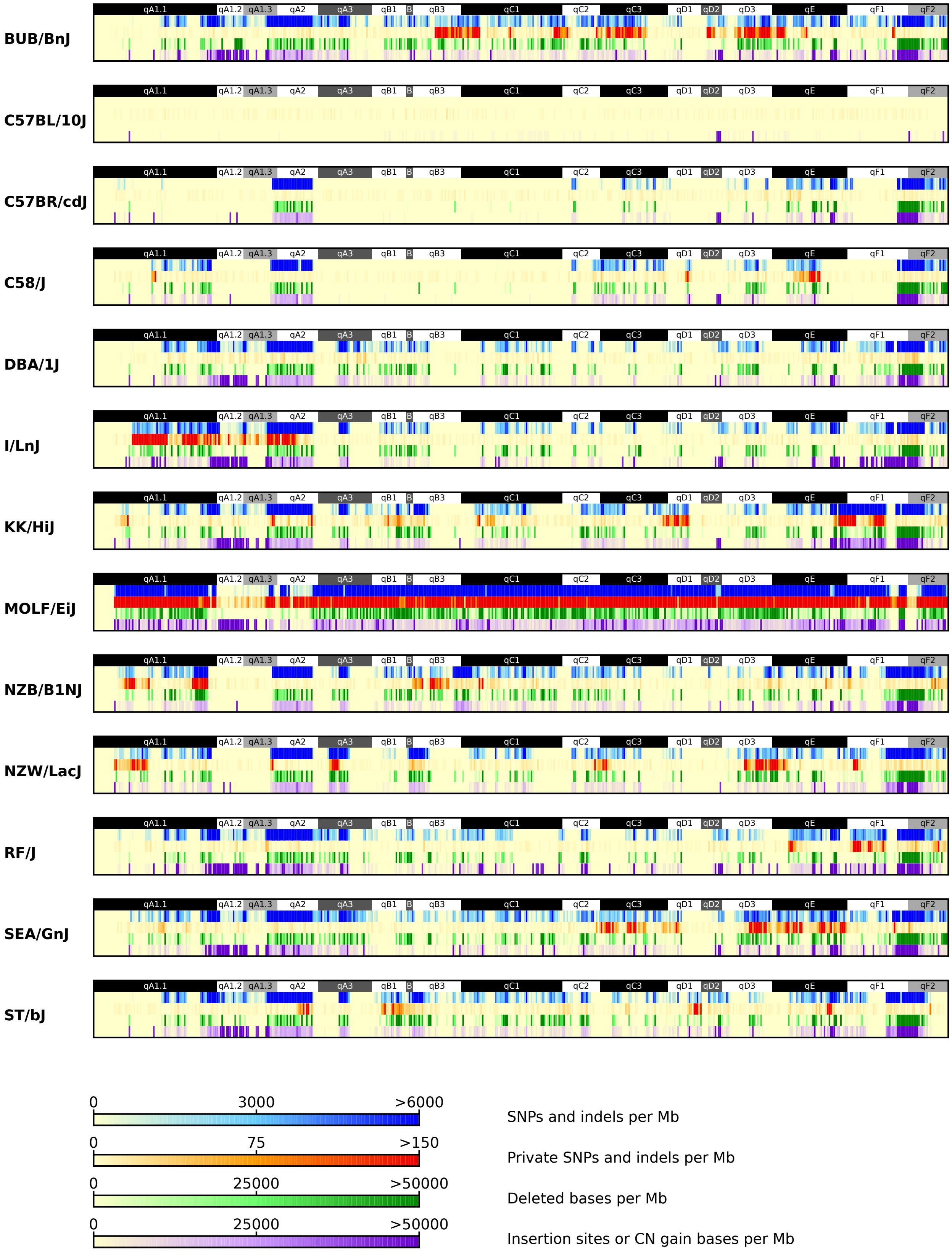

Chromosome 13

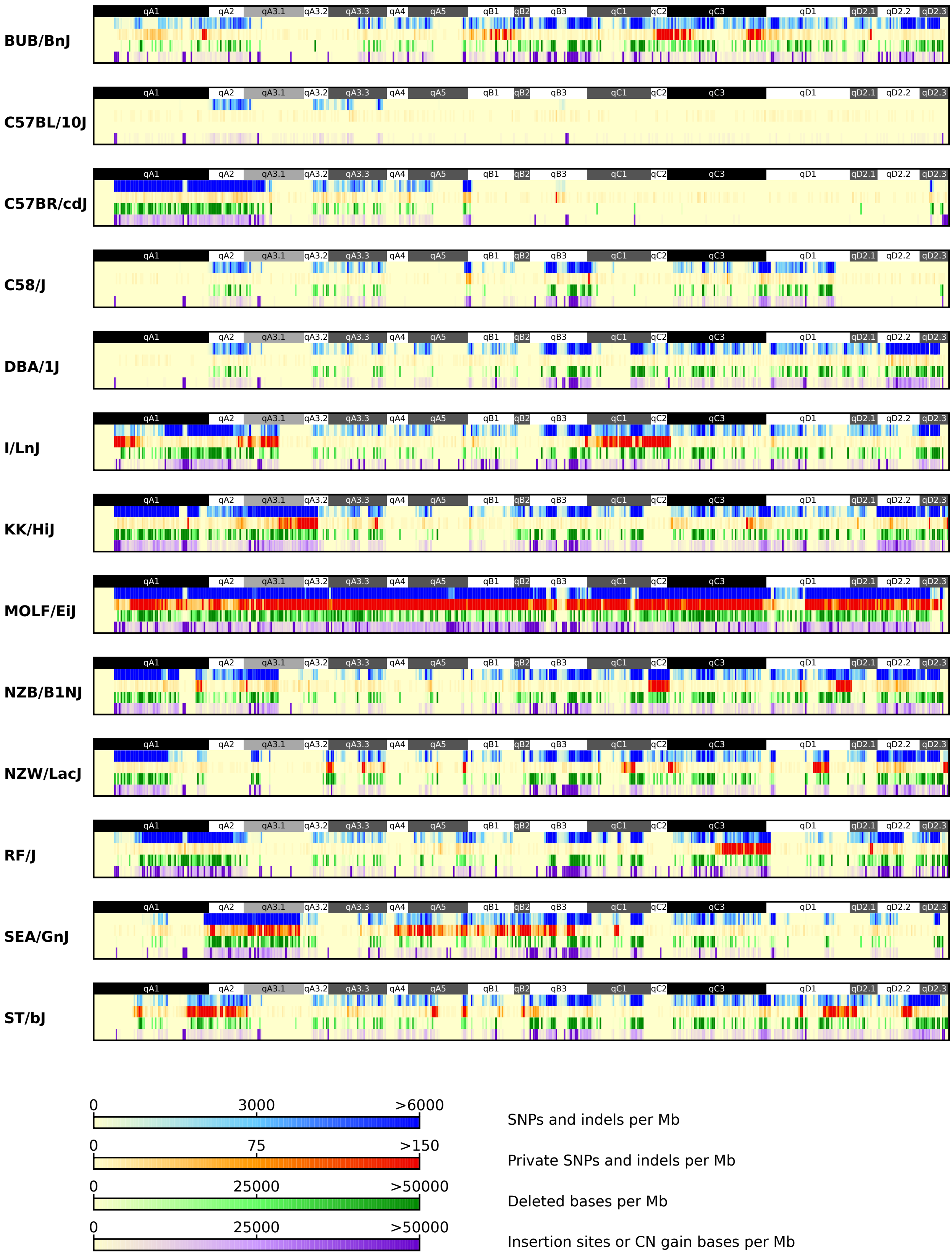

Chromosome 14

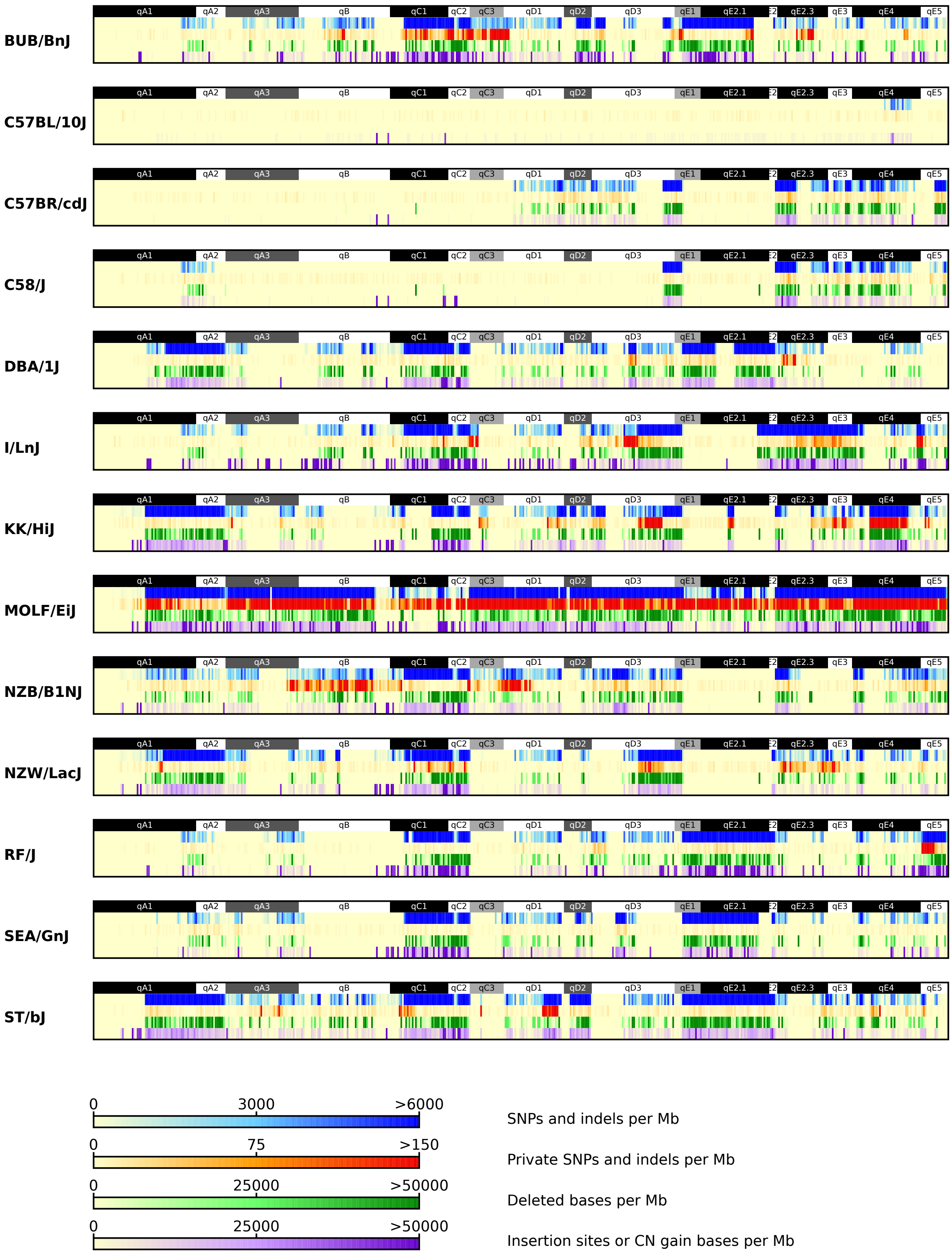

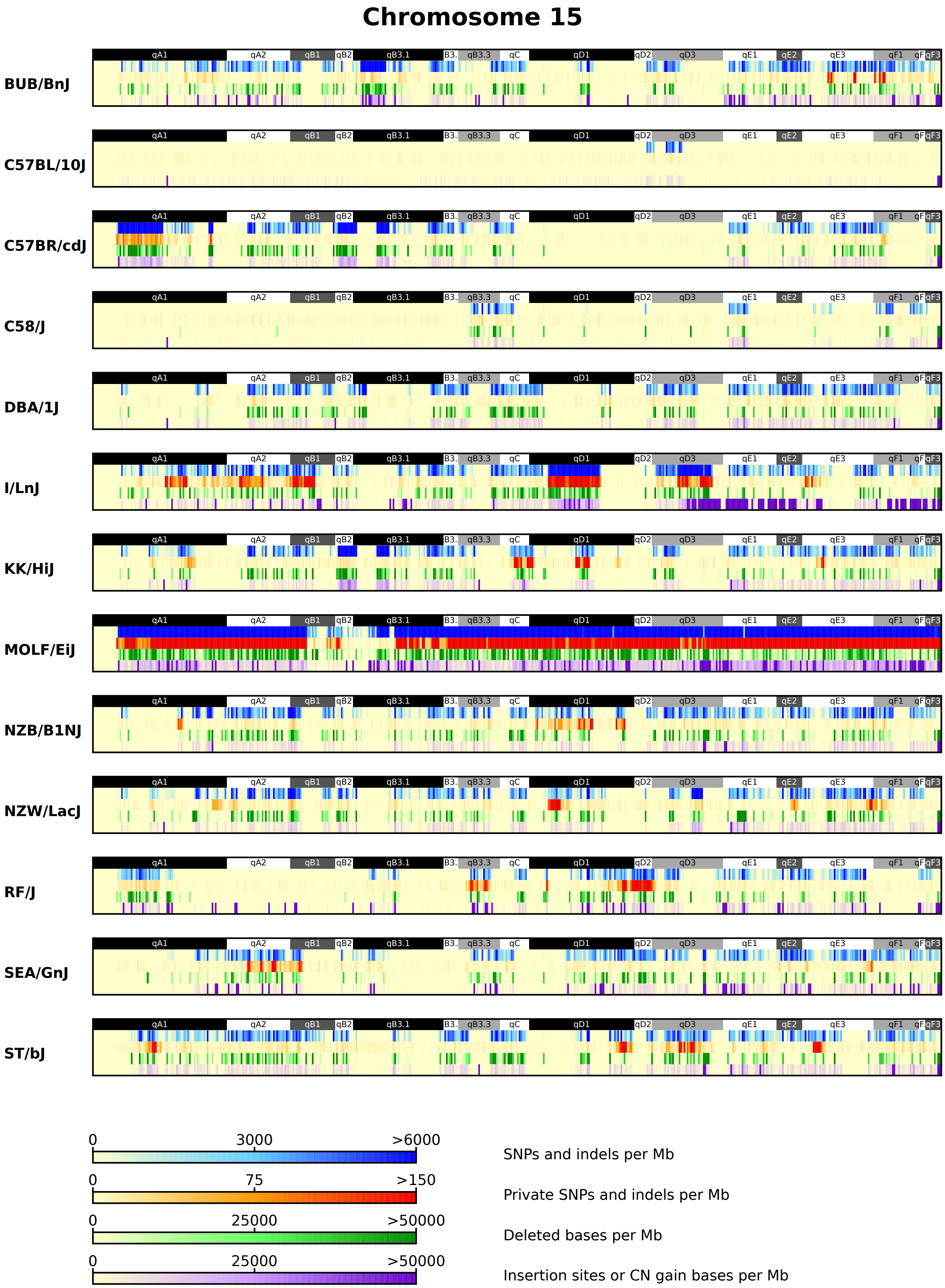
Chromosome 15
BUB/BnJ
C57BL/10J
C57BR/cdJ
C58/J
DBA/1J
I/LnJ
KK/HiJ
MOLF/EiJ
NZB/B1NJ
NZW/LacJ
RF/J
SEA/GnJ
ST/bJ
qA1
qA2
qB1
qB2
qB3.1
B3.
qB3.3
qC
qD1
qD2
qD3
qE1
qE2
qE3
qF1
qF
qF3
0
3000
>6000
SNPs and indels per Mb
0
75
>150
Private SNPs and indels per Mb
0
25000
>50000
Deleted bases per Mb
0
25000
>50000
Insertion sites or CN gain bases per Mb

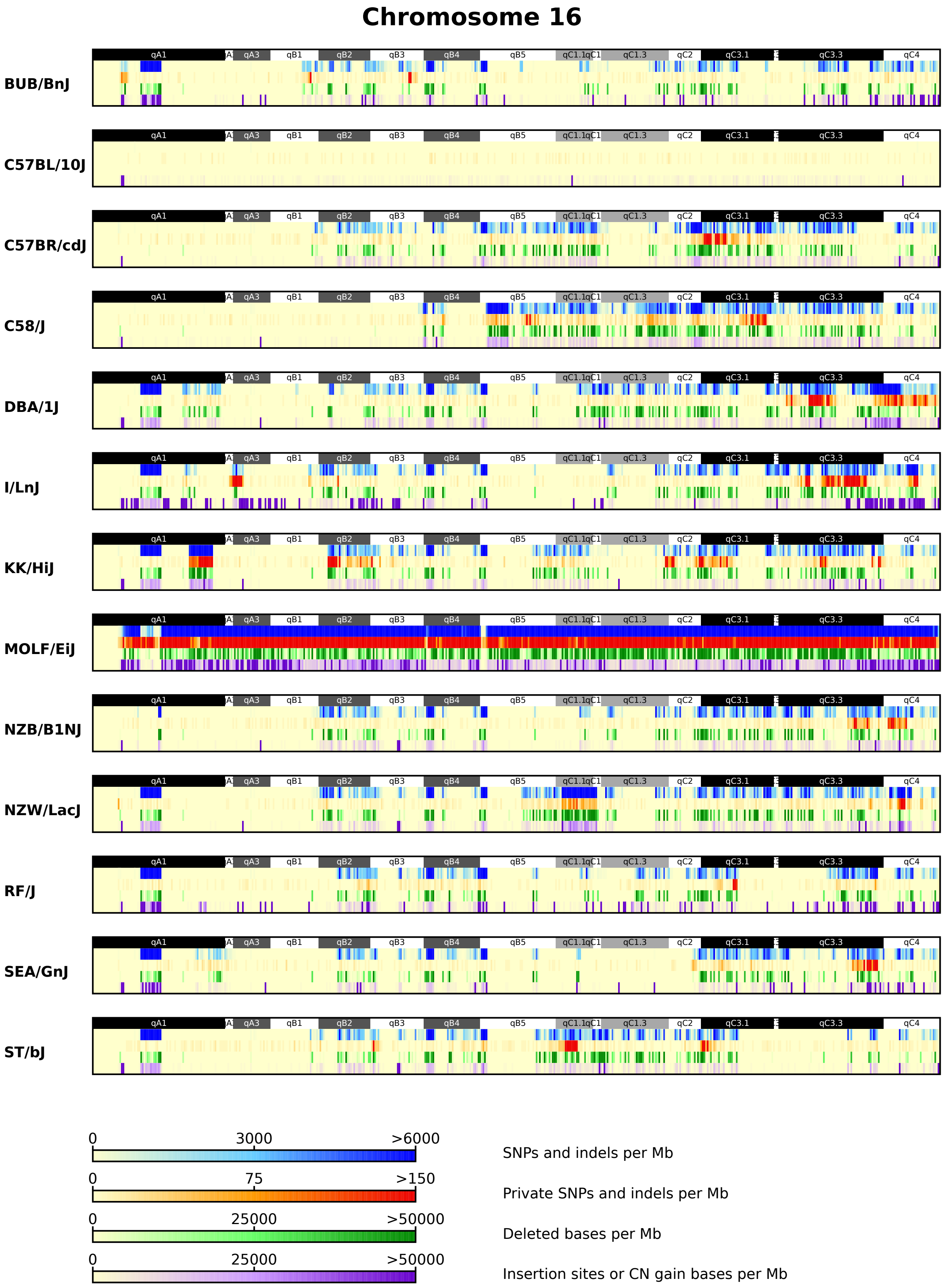

Chromosome 16
qA1
qA3
qB1
qB2
qB3
qB4
qB5
qC1.1
qC1.3
qC2
qC3.1
qC3.3
qC4
BUB/BnJ
C57BL/10J
C57BR/cdJ
C58/J
DBA/1J
I/LnJ
KK/HiJ
MOLF/EiJ
NZB/B1NJ
NZW/LacJ
RF/J
SEA/GnJ
ST/bJ
0
3000
>6000
SNPs and indels per Mb
0
75
>150
Private SNPs and indels per Mb
0
25000
>50000
Deleted bases per Mb
0
25000
>50000
Insertion sites or CN gain bases per Mb

Chromosome 17

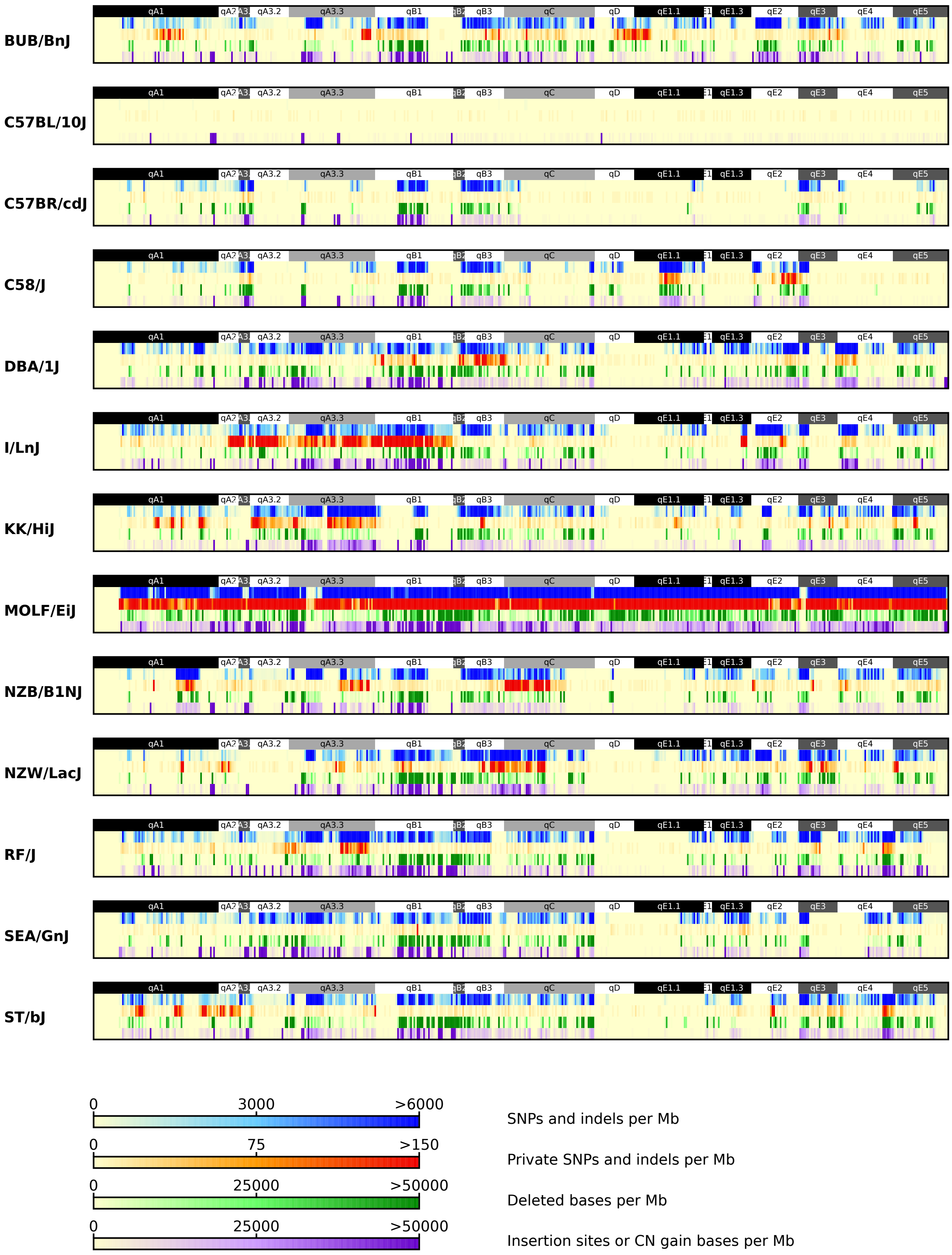

Chromosome 18

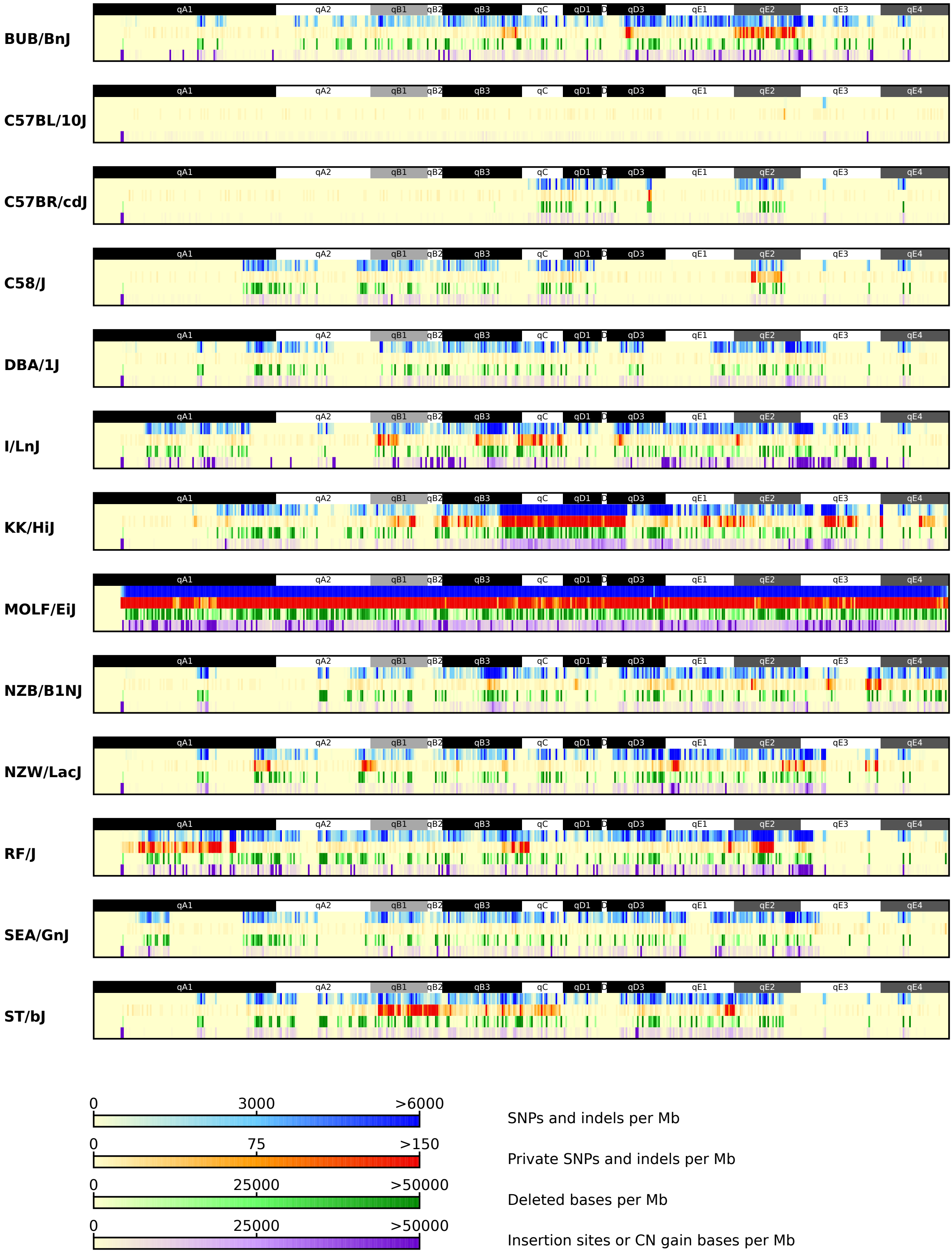

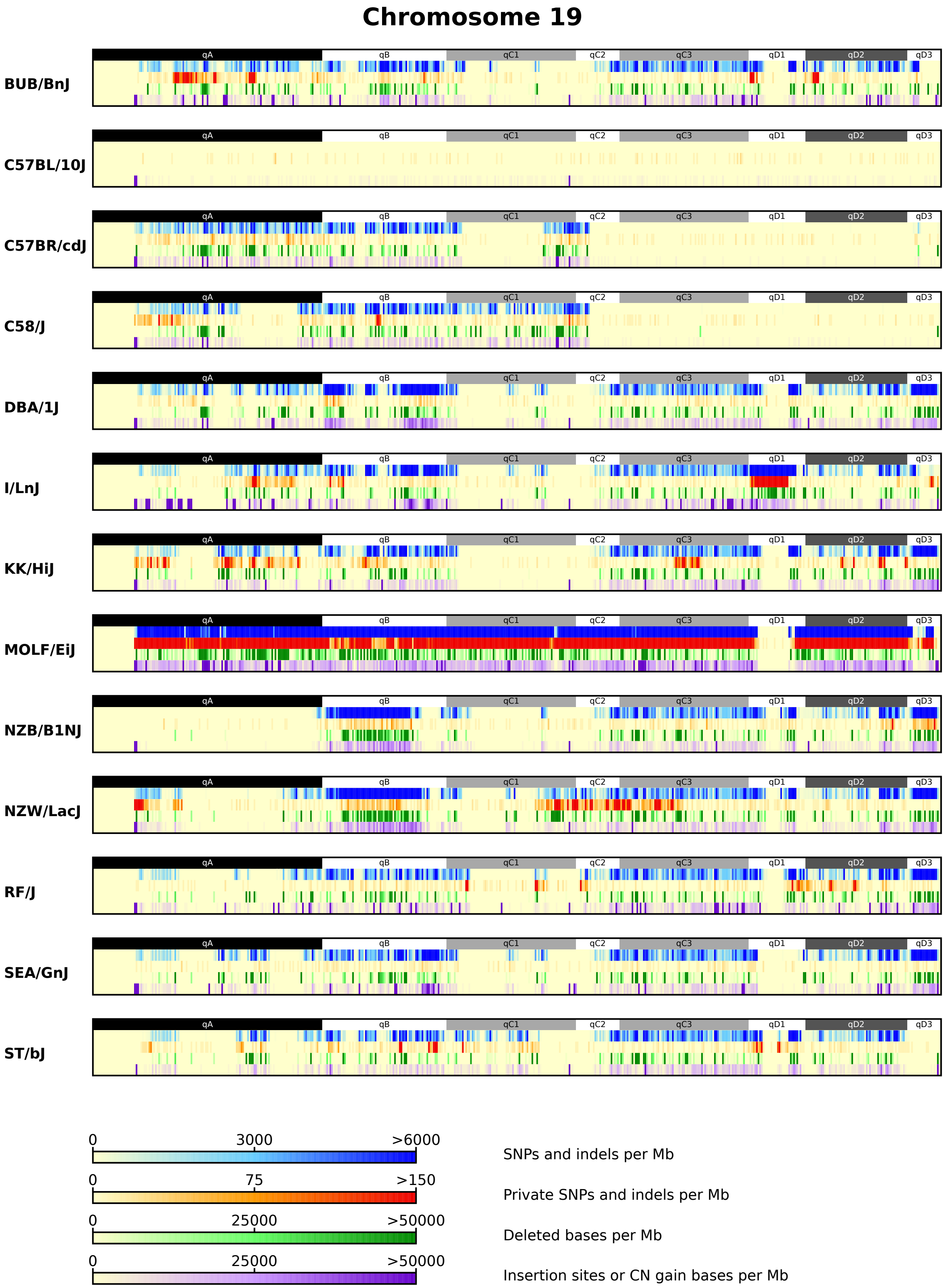
Chromosome 19
BUB/BnJ
C57BL/10J
C57BR/cdJ
C58/J
DBA/1J
I/LnJ
KK/HiJ
MOLF/EiJ
NZB/B1NJ
NZW/LacJ
RF/J
SEA/GnJ
ST/bJ
qA
qB
qC1
qC2
qC3
qD1
qD2
qD3
0
3000
>6000
SNPs and indels per Mb
0
75
>150
Private SNPs and indels per Mb
0
25000
>50000
Deleted bases per Mb
0
25000
>50000
Insertion sites or CN gain bases per Mb

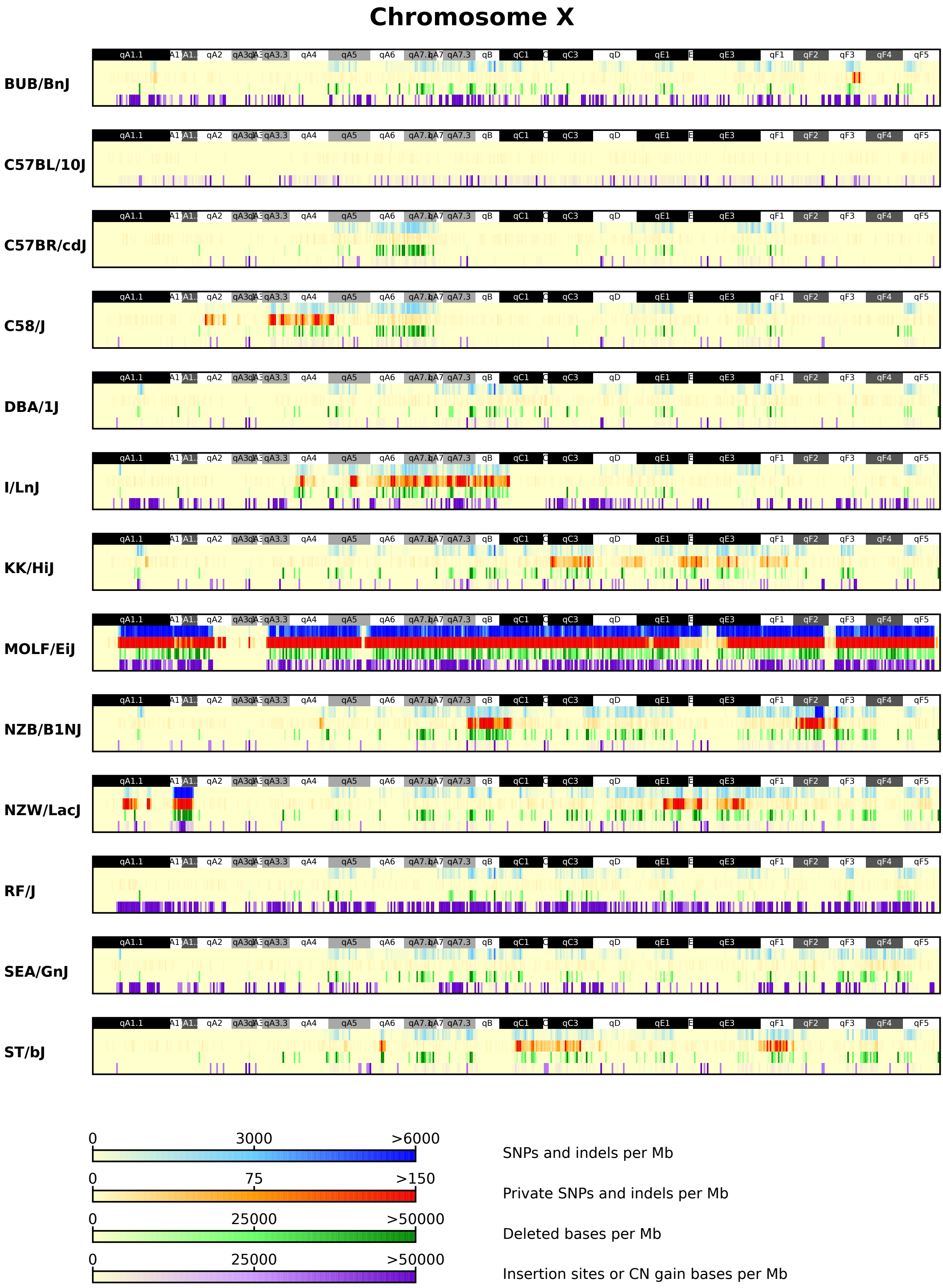

Chromosome X
BUB/BnJ
C57BL/10J
C57BR/cdJ
C58/J
DBA/1J
I/LnJ
KK/HiJ
MOLF/EiJ
NZB/B1NJ
NZW/LacJ
RF/J
SEA/GnJ
ST/bJ
qA1.1
qA2
qA3.3
qA4
qA5
qA6
qA7.3
qB
qC1
qC3
qD
qE1
qE3
qF1
qF2
qF3
qF4
qF5
0
3000
>6000
SNPs and indels per Mb
0
75
>150
Private SNPs and indels per Mb
0
25000
>50000
Deleted bases per Mb
0
25000
>50000
Insertion sites or CN gain bases per Mb

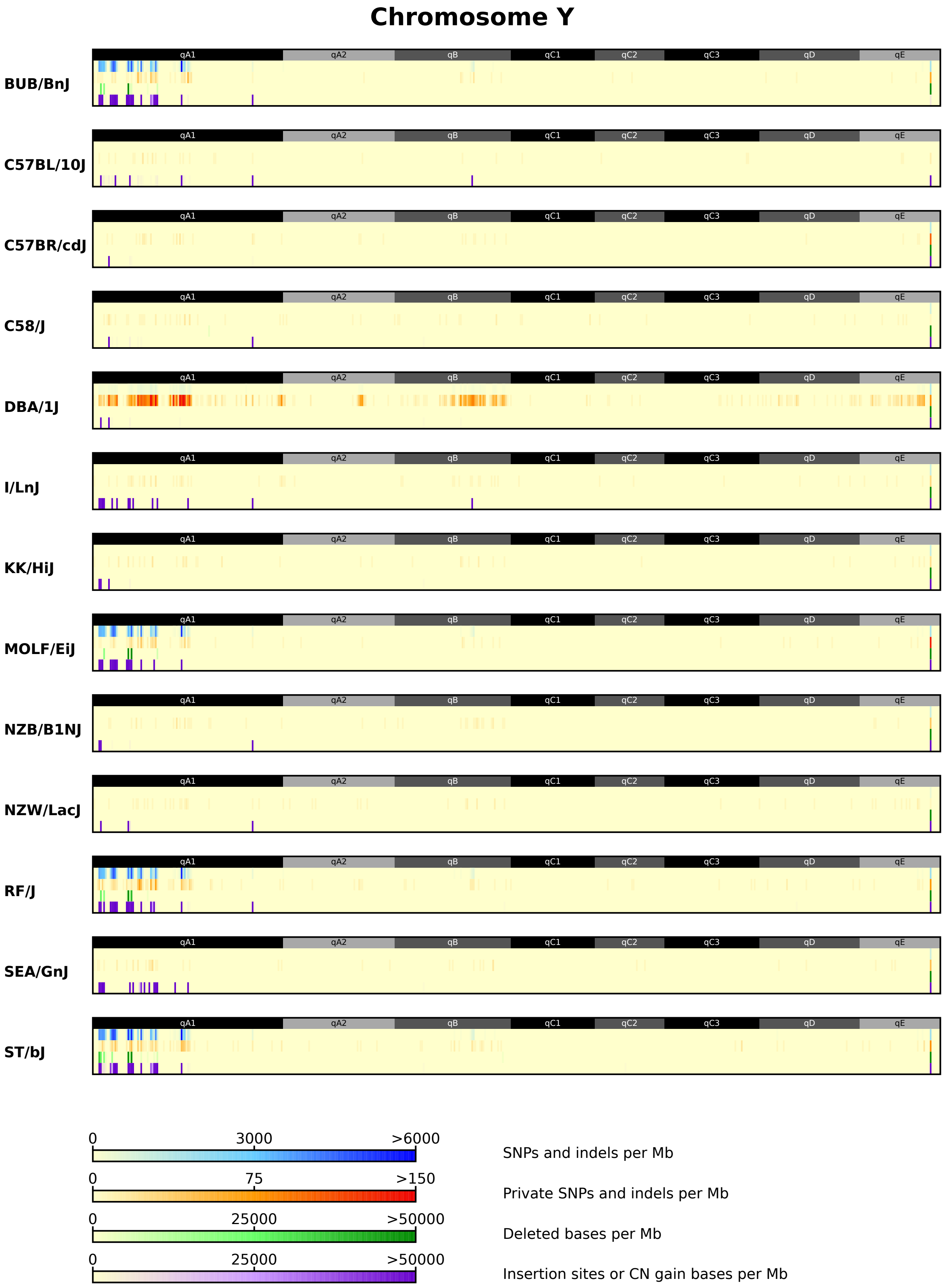
Chromosome Y
BUB/BnJ
C57BL/10J
C57BR/cdJ
C58/J
DBA/1J
I/LnJ
KK/HiJ
MOLF/EiJ
NZB/B1NJ
NZW/LacJ
RF/J
SEA/GnJ
ST/bJ
qA1
qA2
qB
qC1
qC2
qC3
qD
qE
0
3000
>6000
SNPs and indels per Mb
0
75
>150
Private SNPs and indels per Mb
0
25000
>50000
Deleted bases per Mb
0
25000
>50000
Insertion sites or CN gain bases per Mb
